# Lifestyle-Related Factors Contributing to Decline in Knee Extension Strength among Elderly Women: A Cross-Sectional and Longitudinal Cohort Study

**DOI:** 10.1371/journal.pone.0132523

**Published:** 2015-07-15

**Authors:** Narumi Kojima, Miji Kim, Kyoko Saito, Hideyo Yoshida, Yuko Yoshida, Hirohiko Hirano, Shuichi Obuchi, Hiroyuki Shimada, Takao Suzuki, Hunkyung Kim

**Affiliations:** 1 Tokyo Metropolitan Institute of Gerontology, Itabashi-ku, Tokyo, Japan; 2 Yokohama City University Graduate School of Medicine, Kanazawa-ku, Yokohama, Japan; 3 Research Institute, National Center for Geriatrics and Gerontology, 35 Gengo, Morioka-machi, Obu-shi, Aichi, Japan; University of Rome Foro Italico, ITALY

## Abstract

This cross-sectional and 4-year longitudinal cohort study aimed to clarify how various lifestyle-related variables affect knee extension strength in elderly Japanese women. The participants were community-dwelling women (*n* = 575) living in the Itabashi Ward of Tokyo, Japan aged 75–85 years at baseline (in 2008) who returned for a follow-up examination 4 years later (in 2012). Maximum isometric knee extension strength in the dominant leg was measured during comprehensive medical check-ups at baseline and follow-up. Interviews with participants included questions on their history of 11 diseases and lifestyle-related factors such as physical activity as well as dietary, smoking, and drinking habits. Cross-sectional and longitudinal analyses yielded inconsistent results regarding the associations between lifestyle-related factors and knee extension strength. While going out more frequently and regular physical exercise positively affected baseline knee extension strength, they did not affect knee extension strength in the longitudinal analysis. The longitudinal analysis revealed that more frequent intake of soy products or green and yellow vegetables at baseline decreased age-related knee extension strength decline. The inconsistent results from the cross-sectional and longitudinal analyses indicate that conducting both types of analyses is crucial for researching this type of subject. The present study demonstrates that the age-related decline in muscle strength is lower in those who frequently eat soy products or green and yellow vegetables. Thus, recommending higher intake of soy products, and green and yellow vegetables for the elderly might help maintain their muscle health.

## Introduction

As the average life expectancy in Japan is increasing, finding ways to maintain basic levels of activities of daily living (ADL) and instrumental ADL (IADL) among the elderly is important for them to lead independent lives. Muscle strength is well known to directly affect ADL and IADL [1–3]. The prospective study of Rantanen et al. shows that the strength of multiple muscle groups predicts ADL dependence among persons aged ≥75 years; at the 5-year follow-up, those who were in the lowest tertile of muscle strength had a 2- to 3-fold greater risk of becoming ADL-dependent than those in the highest tertile [2]. Furthermore, Kojima et al. report that older women with greater knee extension strength (KES) have a lower prevalence of IADL disability [1].

Several studies use KES as an index of lower-limb function [1], [4–6]. KES declines with aging [7], particularly in the later stage. In a 10-year follow-up study of 120 participants aged 46–78 years at baseline, older participants demonstrated a greater rate of decline in the strength of knee and elbow extensors and flexors [8]. Therefore, minimizing the age-related decline in KES is important for maintaining independence.

Few longitudinal cohort studies have investigated the relationships between lifestyle-related factors and changes in muscle strength. In a 27-year follow-up study of 3,741 men aged 45–68 years at baseline, age as well as a history of stroke, diabetes mellitus, arthritis, coronary heart disease, and chronic obstructive pulmonary disease were factors related to a steeper decline in grip strength [9]. However, the authors did not report any data regarding the influence of modifiable lifestyle-related factors such as eating and exercise habits on muscle strength. A follow-up study of 5,214 women aged 65–91 years at baseline suggests that age, difficulty accomplishing functional tasks, and lower physical activity at baseline are related to a steeper decline in handgrip strength during follow-up [10]. Strenuous work, overweight, smoking, cardiovascular diseases, hypertension, diabetes mellitus, and asthma predicted a greater decline in handgrip strength after 22 years of follow-up of 963 persons aged 30–73 years at baseline in a longitudinal cohort study [11]. Nevertheless, the effects of modifiable lifestyle-related factors such as dietary and exercise habits on age-related changes in KES remain unclear.

Factors that could negatively affect muscle strength in the long term include low levels of physical activity, poor eating habits, and smoking. To our knowledge, no epidemiological study indicates that higher consumption of meat or eggs is beneficial for muscle strength; however, because they are major sources of animal protein, which is an essential material in human muscles, they are likely beneficial. A survey of 2,983 people in the UK indicates that fatty fish consumption positively affects grip strength in both men and women aged 59–73 years [12]. Milk, a good source of quality protein, calcium, and vitamin D, is also expected to have a beneficial effect on muscle health. For example, whey, one of the main proteins in milk, reportedly increases muscle volume when taken regularly as a supplement [13]. Soy products such as tofu, miso, and *natto*, which are routinely consumed by Japanese people, are considered good sources of plant-based proteins. In addition, soy isoflavone and vitamin K, which are found in *natto*, promote bone formation in Japanese women [14–16]. Furthermore, the consumption of green and yellow vegetables and/or fruits is expected to help minimize the age-related decline in muscle strength. A cross-sectional study revealed that daily dietary intake of vitamin C and β-carotene is significantly associated with KES [6]. Alcohol consumption and smoking might also affect muscle strength; for example, a follow-up survey in Finland reports former or current smokers had a greater decline in handgrip strength over 22 years than nonsmokers [11]. Therefore, dietary intake of these food groups, alcohol intake, and smoking habit are factors that potentially affect KES.

Developing a strategy to minimize muscle strength decline among the elderly should be a research priority. In order to develop measures to prevent excessive decline in muscle strength during old age, it is crucial to study how various modifiable lifestyle-related factors affect age-related changes in muscle strength. Therefore, this retrospective cohort study investigated which modifiable lifestyle-related factors have beneficial or detrimental effects on the age-related decline in KES among Japanese community-dwelling women aged 75 or older.

## Methods

### Study Population

The study population consisted of women who participated in a comprehensive health check-up conducted by the Tokyo Metropolitan Institute of Gerontology in 2008 and who returned for a follow-up in October 2012. During the initial check-up in October or November 2008, 1,288 community-dwelling women from the Itabashi Ward of Tokyo were randomly selected and recruited. Their age ranged between 74 and 85 years (mean ± standard deviation: 78.51 ± 2.69 years). Invitation letters were sent to the 1,288 participants of the 2008 check-up (the local government approved using a list of addresses for the residents), and 575 participants (44.6%) returned for follow-up in 2012. The remaining 713 (55.4%) people did not participate because of death (n = 39); refusal to participate, schedule conflict, or inability to attend (n = 606); or unknown reasons (n = 68).

### Ethics statement

The scientific purpose of the study was clearly explained to the participants at enrolment and follow-up. Only data for participants who provided written informed consent were used in this study. This research complies with the ethical rules for human experimentation stated in the Declaration of Helsinki [17]. The study was approved by the Ethics Committee of the Tokyo Metropolitan Institute of Gerontology. No monetary reward was given to the participants, but each participant later received a letter of feedback describing her health status with easily understandable graphs and explanations.

### Measurement of knee extension strength

Isometric KES (in N) was measured in the dominant leg using a hand-held dynamometer (μTas F-1, ANIMA, Chofu, Japan) incorporated into a custom-made frame. Each participant first sat on the horizontal surface of the custom-made frame with her lower legs dangling and knees flexed at 90 degrees; then, she practiced pressing against the tester’s hand until she understood how to execute maximum isometric contraction of the quadriceps. The sensor of the dynamometer was placed 5 cm above the top of the lateral malleolus. The participant was then instructed to exert maximal knee extension force, receiving verbal encouragement as reinforcement. We conducted 2 trials with an interval of approximately 30 seconds. The greater value of the 2 trials was used for analysis. Participants who had been diagnosed with a serious medical problem (e.g., systolic blood pressure >180 mmHg, diastolic blood pressure >110 mmHg, or a history of heart attack or cerebral stroke within the past 6 months) were excluded from the KES test.

### Interviews and variables

During the baseline check-up, the participants were asked closed-ended questions about lifestyle and health status. The interview included items on going out, walking, light exercise, regular exercise and sports, alcohol intake, smoking, intake frequencies of 10 food groups (i.e., seafood, meat, eggs, milk, soy products, green or yellow vegetables, seaweed, potatoes, fruits, and oils and fats), and cohabitation status as well as history of hypertension, stroke, heart disease, diabetes mellitus, hyperlipidemia, osteoporosis, anemia, asthma, chronic obstructive pulmonary disease, hip osteoarthritis, and gonarthrosis.

To evaluate each participant’s habitual dietary patterns, the 10 food groups mentioned above were selected from the 15 original food groups, excluding the 5 food groups that include traditionally eaten staples (e.g., rice, miso soup, pickled vegetables, bread, and noodles) [18]. The questionnaire included examples of specific foods for each category to assist with appropriate selection of the food groups by the interviewers and participants. Examples of seafood included raw fish and all kinds of fish or clam products. The meat category included meat and all meat products. Eggs included chicken or quail eggs but not fish spawn. Milk included only pure milk, excluding milk flavored with coffee or fruit. Soy products were foods made from soybeans, such as tofu and *natto*, which is a traditional Japanese food made of fermented soybeans. Green and yellow vegetables were explained as vegetables that have dark colors, including carrot, spinach, pumpkin, and tomato. The seaweed category included both raw and dried products. Because it was determined that the potato category obviously consisted of white potato, sweet potato, and taro—all of which are consumed by Japanese people—a detailed explanation was not provided. Fruits included all fruits, both fresh and canned. Tomatoes were specified as vegetables, because some Japanese people consider them fruits. Oils and fats included butter, margarine, and all other kinds of oils used for cooking.

While some of the questions were multiple choice (5 response options at most), the responses for all variables were dichotomized for the analysis to ensure that the numbers of positive and negative responders were as similar as possible based on the baseline data. The thresholds based on the baseline data between positive and negative responses were also applied to the follow-up data. “Frequency of going out” had 4 response options (at least once/day, once/2–3 days, once/week, and very rarely), and the participants were classified as “once/2–3 days or less” or “at least once/day” for analysis. Responses to the questions about “strolling or light exercise” and “frequency of participating in such activities/week” were classified as “at least 2–4 days/week” or “once/week or less” for strolling and “at least 5–6 days/week” or “2–4 days/week or less” for light exercise. Regarding the question about “regular exercise and sports,” participants were classified as “yes” or “no.” Participants had 4 response options (almost every day, once/2 days, once or twice/week, or almost never) for the questions on the intake frequency of each food group and were ultimately classified as “once or twice/week or less” or “at least once/2 days” for meat and eggs and “once/2 days or less” or “almost every day” for all other food groups. The Dietary Variety Score (DVS), which is an index of dietary variety introduced by Kumagai et al. [19], was calculated by summing the number of times each participant answered “almost every day” for the intake of each of the 10 food groups [18–20]. The total score ranges from 0 (consuming once/2 days or less for all of the food groups) to 10 (consuming almost every day for all of the food groups). The women were then classified as “DVS ≤5” or “DVS ≥6.” For “alcohol consumption” and “smoking habit,” the participants were classified as “current drinker/smoker” or “non-drinker (never or ex-drinker)/non-smoker (never or ex-smoker).” The response options for the history of the previously listed diseases were “no history” or “with history.” Participants who had a history of a disease were also asked about the current disease status; participants were ultimately classified as “currently negative” or “currently positive.”

### Statistical analysis

Somatometric parameters and KES are presented as population means ± standard deviations. Changes of these parametric variables over 4 years were examined using paired *t*-tests. The population status of each lifestyle-related variable is presented as the number of positive/negative responses. The statistical significance of changes in the ratios of these non-parametric variables was examined using the McNemar test.

A cross-sectional analysis was conducted to examine the relationships between the lifestyle-related variables and KES at baseline in the 575 women who also participated in the follow-up survey. For each lifestyle-related variable, we compared KES between those with positive and negative responses at baseline by using ANCOVA adjusted for baseline age and current status of all diseases, with each disease included as a separate variable.

We also conducted a longitudinal analysis to examine the associations between baseline lifestyle-related variables and changes in KES over 4 years. For each variable, we compared the changes in KES between those with positive and negative responses at baseline by using ANCOVA adjusted for baseline age, KES, and the status of all diseases, with each disease included as a separate variable.

All statistical analyses were conducted using PASW Statistics 18 (IBM Corp., Armonk, NY, USA). The results were regarded statistically significant when the *P*-value was <0.05.

## Results

### Participant characteristics at baseline and follow-up

The age of the 575 participants ranged between 75 and 85 years (78.07 ± 2.56) in 2008 and between 78 and 89 years (82.07 ± 2.55) in 2012 (Table 1). At baseline, the participants who did not return for the 2012 check-up were significantly older (78.86 ± 2.74 versus 78.07 ± 2.56 years, respectively) and had lower KES (192.36 ± 48.60 versus 205.95 ± 53.36 N, respectively) than those who returned. Both average the height and weight of the 575 final study participants decreased significantly over the 4 years of the study (*P* < 0.001, Table 1). No changes in body mass index or %body fat were observed. KES decreased significantly by approximately 10% over the course of 4 years.

**Table 1:** Subjects' characteristics at baseline and follow-up at 4 years (n = 575).

Variables	Baseline		Follow-Up		P-Value
	Mean	SD	Mean	SD	
Measured values					
Age	78.07	±2.56	82.07	±2.55	
Height, cm	148.42	±5.29	147.32	±5.50	P<0.001
Weight, kg	50.20	±7.89	49.33	±8.18	P<0.001
Body mass index, kg/m^2^	22.78	±3.34	22.72	±3.53	0.286
%Body fat	31.94	±4.71	32.07	±7.74	0.528
Knee extension strength, N	205.95	±53.36	186.01	±54.60	P<0.001
	n	(%)	n	(%)	
Physical activities					
Going out (at least once per day)	472	(82.1)	419	(72.9)	P<0.001
Going for a stroll (at least 2–4days per week)	343	(59.7)	371	(64.5)	0.054
Light exercises (at least 5–6days per week)	287	(49.9)	314	(54.6)	0.060
Regular exercises and sports (Yes)	234	(40.7)	201	(35.0)	0.007
Foods & discretionary items					
Seafood (almost every day)	260	(45.2)	238	(41.4)	0.132
Meat (at least once per 2 days)	341	(59.3)	374	(65.0)	0.011
Egg (at least once per 2 days)	369	(64.2)	393	(68.3)	0.071
Milk (almost every day)	357	(62.1)	368	(64.0)	0.375
Soy products (almost every day)	395	(68.7)	358	(62.3)	0.006
Green and yellow vegetables (almost every day)	507	(88.3)	483	(84.0)	0.021
Seaweeds (almost every day)	314	(54.6)	252	(43.8)	P<0.001
Potatoes (almost every day)	248	(43.1)	211	(36.7)	0.326
Fruits (almost every day)	500	(87.0)	487	(84.7)	0.006
Oils and fats (almost every day)	291	(50.6)	345	(60.0)	0.039
Dietary variety score (≥6 points)	290	(50.5)	267	(46.4)	0.073
Drinking (Current drinker)	149	(25.9)	125	(21.7)	0.006
Smoking (Smoker)	22	(3.8)	15	(2.6)	0.039
Diseases					
Hypertension (positive)	306	(53.2)	365	(63.5)	P<0.001
Stroke (positive)	27	(4.7)	36	(6.3)	0.124
Heart disease (positive)	106	(18.4)	123	(21.4)	0.075
Diabetes mellitus (positive)	46	(8.0)	48	(8.3)	0.791
Hyperlipidemia (positive)	225	(39.1)	246	(42.9)	0.083
Osteoporosis (positive)	160	(27.8)	215	(37.5)	P<0.001
Anemia (positive)	10	(1.7)	21	(3.7)	0.043
Asthma (positive)	18	(3.1)	23	(4.0)	0.267
COPD (positive)	1	(0.2)	3	(0.5)	0.625
Osteoarthritis of hip (positive)	11	(1.9)	18	(3.1)	0.143
Gonarthritis (positive)	126	(22.0)	156	(27.1)	0.005

SD = standard deviation, COPD = chronic obstructive pulmonary disease, P-Values were outcomes of paired t-tests for continuous variables, and of McNemar tests for binary variables.

The data for physical activities and other lifestyle factors showed that the participants generally became less active 4 years after baseline (Table 1). The proportion of women who went out at least once/day or participated in regular exercise and sports decreased significantly (*P* < 0.001, *P* = 0.007, respectively). The proportions of women who drank alcohol or smoked decreased by 4.2% and 1.2%, respectively (Table 1).

Regarding intakes of the different food groups, the proportion of women who consumed some of the food groups (i.e., meats, and oils and fats) more frequently (≥once per 2 days for meat and eggs, almost every day for other food groups) increased significantly over the course of 4 years (Table 1). The proportion of participants who ate meat at least once/2 days increased significantly by 5.7% (from 59.3% at baseline to 65.0% at follow-up; *P* = 0.011). The proportion of those who ate oils and fats almost every day increased significantly by 9.4% (50.6–60.0%; *P* = 0.039). In contrast, the proportions of women who ate soy products, green or yellow vegetables, seaweed, or fruits almost every day decreased significantly (all *P* < 0.05; Table 1). The proportions of women who had hypertension, osteoporosis, anemia, or gonarthrosis increased significantly over the 4 years (all *P* < 0.05; Table 1).

### Cross-sectional analysis of knee extension strength at baseline

Cross-sectional analysis of the baseline data showed that some factors related to physical activity were significantly associated with KES at baseline (Table 2). The mean KES was higher in those who went out once/day or more or participated in regular exercise and sports than those who did not. We found no significant relationship between KES and other lifestyle-related factors including the frequency of walking, frequency of food intake of the studied food groups, DVS, alcohol intake, and smoking (Table 2).

**Table 2:** Result of the cross-sectional analysis showing the average knee extension strength according to binarized baseline lifestyle factors.

	Mean	SE	P-Value
Physical activities			
Going out			
Once per 2–3days or less (N = 98)	191.09	(5.24)	
At least once per day (N = 448)	209.62	(2.44)	.001
Going for a stroll			
1day per week or less (N = 214)	205.52	(3.56)	
At least 2–4days per week (N = 332)	206.77	(2.86)	.784
Light exercises			
2–4days per week or less (N = 272)	201.54	(3.15)	
At least 5–6days per week (N = 274)	211.02	(3.15)	.035
Regular exercises and sports			
No (N = 322)	199.99	(2.89)	
Yes (N = 224)	215.26	(3.47)	.001
Foods & discretionary items			
Seafood			
Once per 2 days or less (N = 299)	204.98	(3.01)	
Almost every day (N = 247)	207.87	(3.32)	.521
Meat			
1–2 times per week or less (N = 228)	203.31	(3.46)	
At least once per 2 days (N = 318)	208.41	(2.92)	.263
Egg			
1–2 times per week or less (N = 197)	200.51	(3.73)	
At least once per 2 days (N = 349)	209.55	(2.79)	.055
Milk			
Once per 2 days or less (N = 204)	203.45	(3.67)	
Almost every day (N = 342)	207.96	(2.82)	.333
Soy products			
Once per 2 days or less (N = 173)	201.72	(3.98)	
Almost every day (N = 373)	208.39	(2.69)	.168
Green and yellow vegetables			
Once per 2 days or less (N = 66)	203.52	(6.43)	
Almost every day (N = 479)	206.64	(2.38)	.650
Seaweeds			
Once per 2 days or less (N = 248)		(3.31)	
Almost every day (N = 298)	209.31	(3.02)	.139
Potatoes			
Once per 2 days or less (N = 311)		(2.97)	
Almost every day (N = 235)	208.22	(3.43)	.457
Fruits			
Once per 2 days or less (N = 71)		(6.22)	
Almost every day (N = 475)	207.24	(2.38)	.270
Oils and fats			
Once per 2 days or less (N = 271)		(3.18)	
Almost every day (N = 275)	205.53	(3.15)	.736
Dietary variety score			
DVS ≤ 5 points (N = 269)	198.11	(2.07)	
DVS ≥ 6 points (N = 276)	199.17	(2.00)	.714
Drinking			
Non-drinker (N = 404)	204.08	(2.58)	
Current drinker (N = 142)	212.50	(4.37)	.099
Smoking			
Non-smoker (N = 525)		(2.26)	
Smoker (N = 21)	220.99	(11.41)	.189

SE; Standard Error, Analyses of covariance were applied incorporating baseline age, and baseline status of all the diseases (hypertension, stroke, heart disease, diabetes mellitus, hyperlipidemia, osteoporosis, anemia, asthma, chronic obstructive pulmonary disease, hip osteoarthritis, gonarthrosis) as covariates.

### Longitudinal analysis of the effects of lifestyle-related factors on knee extension strength

Longitudinal analysis showed that except for 3 food groups, no lifestyle-related variables at baseline were related to changes in KES over 4 years (Table 3). Those who ate seafood almost every day had a significantly greater decrease (1.5 times) in KES (24.68 N) than those who ate seafood once/2 days or less (16.88 N) (*P* = 0.022). In contrast, the intake of soy products and green or yellow vegetables had a beneficial effect on KES. The decrease of KES in participants who ate soy products almost every day (17.87 N) was approximately 69% of that in those who ate soy products once/2 days or less (26.06 N) (*P* = 0.026), and the decrease of KES in participants who ate green or yellow vegetables almost every day (18.82 N) was approximately 60% of that in those who ate these vegetables once/2 days or less (31.46 N; *P* = 0.015; Table 3).

**Table 3:** Result of the longitudinal analysis showing the effect of baseline lifestyle factors on decline in muscle strength.

	Mean	SE	P-Value
Physical activities			
Going out			
Once per 2–3days or less (N = 95)	-19.87	(4.05)	
At least once per day (N = 439)	-20.51	(1.86)	.886
Going for a stroll			
1day per week or less (N = 207)	-22.53	(2.70)	
At least 2–4days per week (N = 327)	-19.04	(2.15)	.314
Light exercises			
≤2–4days per week (N = 266)	-19.94	(2.39)	
At least 5–6days per week (N = 268)	-20.86	(2.39)	.787
Regular exercises and sports			
No (N = 314)	-21.61	(2.22)	
Yes (N = 220)	-18.67	(2.66)	.404
Foods & discretionary items			
Seafood			
Once per 2 days or less (N = 292)	-16.88	(2.26)	
Almost every day (N = 242)	-24.68	(2.50)	.022
Meat			
1–2 times per week or less (N = 223)	-18.88	(2.62)	
At least once per 2 days (N = 311)	-21.48	(2.21)	.451
Egg			
1–2 times per week or less (N = 192)	-19.37	(2.84)	
At least once per 2 days (N = 342)	-20.98	(2.12)	.654
Milk			
Once per 2 days or less (N = 199)	-22.31	(2.78)	
Almost every day (N = 335)	-19.26	(2.13)	.388
Soy products			
Once per 2 days or less (N = 165)	-26.06	(3.04)	
Almost every day (N = 369)	-17.87	(2.02)	.026
Green and yellow vegetables			
Once per 2 days or less (N = 64)	-31.46	(4.85)	
Almost every day (N = 469)	-18.82	(1.78)	.015
Seaweeds			
Once per 2 days or less (N = 240)		(2.52)	
Almost every day (N = 294)	-18.10	(2.27)	.135
Potatoes			
Once per 2 days or less (N = 302)		(2.25)	
Almost every day (N = 232)	-18.08	(2.57)	.237
Fruits			
Once per 2 days or less (N = 70)		(4.68)	
Almost every day (N = 464)	-19.18	(1.80)	.068
Oils and fats			
Once per 2 days or less (N = 264)		(2.41)	
Almost every day (N = 270)	-22.30	(2.37)	.256
Dietary variety score			
DVS ≤5 points (N = 261)	-22.73	(2.42)	
DVS ≥6 points (N = 272)	-18.06	(2.37)	.171
Drinking			
Non-drinker (N = 395)	-20.06	(1.96)	
Current drinker (N = 139)	-21.33	(3.31)	.742
Smoking			
Non-smoker (N = 514)	-20.25	(1.71)	
Smoker (N = 20)	-24.14	(8.77)	.664

SE; Standard Error, Analyses of covariance were applied incorporating baseline age, baseline knee extensor strength, and baseline status of all the diseases (hypertension, stroke, heart disease, diabetes mellitus, hyperlipidemia, osteoporosis, anemia, asthma, chronic obstructive pulmonary disease, hip osteoarthritis, and gonarthrosis) as covariates.

## Discussion

We conducted both cross-sectional and longitudinal analyses of the factors associated with KES on a cohort of elderly women in Japan. However, the associations between lifestyle-related factors and KES were inconsistent between analyses. Our cross-sectional analysis showed that women who go out or exercise regularly had a higher KES than those who did not. However, these variables were not significantly associated with changes in KES in the longitudinal analysis. Therefore, the significant association between physical activity-related lifestyle variables and KES at baseline might merely reflect the fact that women with strong legs were able to perform physical activities with fewer limitations.

The intake frequencies of seafood, soy products, and green or yellow vegetables, which were not associated with KES at baseline, were associated with changes in KES over 4 years. The routine intake of seafood negatively affected KES, while those of soy products and green and yellow vegetables had beneficial effects. The apparent discrepancy between the results of the 2 analyses indicates that conducting a cross-sectional analysis alone is insufficient for investigating the influences of lifestyle-related factors on muscle strength.

We did not expect that variables found to be associated with KES in the cross-sectional analysis (i.e., going out, and regular exercise and sports) would not show associations in the longitudinal analysis. Several intervention studies have established that exercise is effective for maintaining or even increasing muscle strength in the elderly [21–23]. One possible reason why our data do not seem to support the effect of physical activities on muscle strength is that some participants did not maintain their baseline physical activity level. However, an additional comparison between those who reported consistently going out and those who consistently reported not going out yielded no significant association with a change in KES (P = 0.307; data not shown). Therefore, the consistency of physical activity does not seem to be critical for maintaining muscle strength among elderly women. Although our study failed to find an association of regular physical activity with KES, the beneficial effect of exercise on muscle strength cannot be denied.

The decline in KES was greater in women who ate seafood almost every day at baseline. However, this result may not be very robust, because the KES at baseline was slightly higher in women who ate seafood almost every day. Several studies indicate that regular seafood intake contributes to maintaining muscle strength [12,24,25]. For example, a cross-sectional study in the UK shows that fatty fish consumption positively influences grip strength in older people [12]. Rats fed Alaska pollock protein exhibit greater fast-twitch muscle hypertrophy than rats fed casein [24]. In a study conducted in older people, leg extension power was positively associated with the serum concentration of an active vitamin D derivative, which is abundant in seafood [25]. However, methyl mercury, a compound found in seafood, might negatively affect muscle strength. Accordingly, frequent intake of seafood with high levels of mercury in coastal villages is associated with a risk of toxicity, including weakened muscles [26,27]. However, because our study was conducted in Tokyo where no such cases have been reported, seafood intake does not seem to have affected muscle strength. Therefore, a study with a larger cohort seems necessary to clarify the effect of daily seafood intake on muscle strength.

The 4-year decline in KES was smaller in those who ate soy products almost every day than those who ate soy products once/2 days or less. Although we could not find direct evidence supporting the beneficial effect of soy products on muscle health in the literature, several studies suggest that some ingredients found in soy products, such as vitamin K_2_ and isoflavone, improve bone metabolism [16–14,28]. In a study of 4 groups of participants eating different amounts of *natto* (rich in vitamin K_2_) for 1 year, the risk of a reduction in bone formation markers in the group that ate *natto* most frequently was 0.07 times of that in the group that ate no *natto* [14]. Another prospective study of middle-aged Japanese women shows that 24 weeks of soy isoflavone supplementation increases bone mineral density [15]. A placebo-controlled study shows that menopausal women treated with isoflavone tablets exhibited a significant decrease in urinary excretion of urinary deoxypyridinoline, a specific biomarker of bone resorption [16]. An analysis of the association of regional differences in *natto* intake and hip fracture incidence revealed that fractures are less prevalent in regions where *natto* consumption is prevalent due to local culture [28]. The daily intake of soy products in our study may have contributed to the maintenance of muscle strength through a reduction of the incidence of fractures and resulting inactivity.

However, the questionnaire items about food intake used in the present study were not detailed enough to establish concrete data about the dietary habits of elderly people. Because our study was based on a retrospective analysis of existing data, we were unable to use very detailed questions. Therefore, studies using more detailed items about food intake aiming to understand how dietary habits affect elderly people’s muscle strength seem necessary to make suggestions regarding the diet of elderly people for maintaining muscle strength.

The smaller decline of KES in women who ate green or yellow vegetables almost every day may be explained by the antioxidant effects of carotenoids and vitamin C. Recent epidemiological studies in community-dwelling elderly show that low serum concentrations of carotenoids or vitamin C are associated with low muscle strength [4,6,29–31]. A cross-sectional study revealed that daily dietary intake of vitamin C and β-carotene is significantly associated with KES [6]. A longitudinal observational study of people >64 years old further shows that those in the lowest quartile of total plasma carotenoid level have a significantly higher risk of muscle weakening 6 years later than those in the highest quartile [5]. Concordant with these studies, the present study corroborates the beneficial effect of the daily intake of green or yellow vegetables on age-related decline in muscle strength.

Contrary to our expectation, the intake frequency of meat at baseline did not influence changes in muscular strength. In a study of 1,844 Japanese senior citizens, those with the lowest initial density of serum albumin, which is processed from other proteins within the human body, had the highest incidence of ADL disability 12 years later [32]. In contrast, a longitudinal study in healthy aged men suggests that a low albumin concentration does not predict a decline in muscle strength [33]. Furthermore, a low baseline concentration of serum albumin failed to predict a decline in grip strength over 2 years in a study of frail elderly participants [34]. Another longitudinal study in Japanese elderly shows that a higher intake frequency of animal protein is associated with a lower risk of decline in functional capacity only in men [35]. As the association between protein intake and muscle health remains controversial, more cohort studies are needed to elucidate how meat intake affects muscle strength in the elderly.

This study has several limitations. First, we could not determine the amounts of each food consumed at the time of the baseline survey; instead, the questionnaire collected information about the number of days in a week that a given food was eaten. Nevertheless, we expect that the results of the questionnaire are at least generally related with the actual amounts of food eaten. Second, the data regarding lifestyle factors were only based on the answers to questions at a single time point. Therefore, it is uncertain whether a particular subject kept eating soy products, for example, at the same frequency for 4 years. Third, the data about physical activity levels were collected by interview. Although an objective measurement of physical activity is more appropriate for evaluating lifestyle, the retrospective design forced us to use the data obtained through the interviews.

In conclusion, the cross-sectional and longitudinal analyses yielded inconsistent results regarding the associations of lifestyle-related factors with KES. This inconsistency suggests that conducting both types of analyses is crucial. Because our study demonstrated that the age-related decline in muscle strength was lower in those who frequently ate soy products or green and yellow vegetables, recommending higher intakes of these foods might be a useful measure for protecting the muscle health of the elderly.

## Supporting Information

S1 Dataset Dataset containing all relevant data of the participants in 2008 and 2012. (XLSX)
